# Familial risk of Wolff–Parkinson–White syndrome: a nationwide family study in Sweden

**DOI:** 10.1093/europace/euaf285

**Published:** 2025-11-08

**Authors:** Fabrizio Ricci, Mirnabi Pirouzifard, Kristian Galanti, Jan Sundquist, Kristina Sundquist, Richard Sutton, Artur Fedorowski, Bengt Zöller

**Affiliations:** Department of Neuroscience, Imaging and Clinical Sciences, ‘G. D’Annunzio’ University of Chieti-Pescara, Via Luigi Polacchi, 11 - 66100 Chieti, Italy; Institute for Advanced Biomedical Technologies, ‘G. D’Annunzio’ University of Chieti-Pescara, Via Luigi Polacchi, 11 - 66100 Chieti, Italy; Department of Clinical Sciences, Lund University, Jan Waldenströms gata 35, 214 28 Malmö, Sweden; University Cardiology Division, Heart Department, SS. Annunziata Hospital, ASL 02 Abruzzo, Via dei Vestini, 66100 Chieti, Italy; Center for Primary Health Care Research, Department of Clinical Sciences, Lund University, Malmö, Sweden; University Clinic Primary Care, Skåne University Hospital, Region Skåne, Sweden; Department of Neuroscience, Imaging and Clinical Sciences, ‘G. D’Annunzio’ University of Chieti-Pescara, Via Luigi Polacchi, 11 - 66100 Chieti, Italy; Center for Primary Health Care Research, Department of Clinical Sciences, Lund University, Malmö, Sweden; University Clinic Primary Care, Skåne University Hospital, Region Skåne, Sweden; Center for Primary Health Care Research, Department of Clinical Sciences, Lund University, Malmö, Sweden; University Clinic Primary Care, Skåne University Hospital, Region Skåne, Sweden; Department of Clinical Sciences, Lund University, Jan Waldenströms gata 35, 214 28 Malmö, Sweden; Department of Cardiology, National Heart & Lung Institute, Imperial College, London, United Kingdom; Department of Clinical Sciences, Lund University, Jan Waldenströms gata 35, 214 28 Malmö, Sweden; Department of Cardiology, Karolinska University Hospital and Karolinska Institute, Stockholm, Sweden; Center for Primary Health Care Research, Department of Clinical Sciences, Lund University, Malmö, Sweden; University Clinic Primary Care, Skåne University Hospital, Region Skåne, Sweden

**Keywords:** Wolff–Parkinson–White, Ventricular Pre-excitation, Family risk, Inheritance patterns, Arrhythmias

## Abstract

**Aims:**

Wolff–Parkinson–White (WPW) syndrome is a rare cardiac disorder that predisposes to supraventricular arrhythmias. Prognosis is usually benign, yet there is an increased lifetime risk of sudden death. While typically sporadic, familial clustering has been reported. This study aimed to assess the risk of WPW, arrhythmias, and mortality among siblings of individuals with WPW.

**Methods and results:**

This population-based sibling cohort included 5 338 434 individuals born in Sweden (1932–2018), with 3172 WPW cases identified from the Swedish National Patient Registers. Familial risks among siblings were assessed using incidence rate ratios (IRRs) and adjusted subdistributional hazard ratios (SHRs). Sensitivity analyses excluded syndromic WPW and cases without electrophysiologic procedural confirmation. Although familial occurrence of WPW was exceedingly rare with only 14 of 3172 cases (0.4%; ≈0.0003% of the total population), siblings of affected individuals showed a significantly higher rate of WPW diagnosis (0.121 vs. 0.032 per 1000 person-years; IRR 3.83; 95% CI 2.27–6.46; *P* < 0.001) translating to an almost four-fold higher adjusted risk (SHR 3.79; 95% CI 1.81–7.97; *P* < 0.001). Risks of atrial fibrillation (SHR 1.19; 95% CI 1.05–1.35; *P* < 0.01) and ventricular arrhythmias (SHR 1.84; 95% CI 1.45–2.35; *P* < 0.001) were also higher, whereas all-cause mortality was comparable irrespective of sibling history (HR 1.01; 95% CI 0.92–1.11; *P* = 0.88).

**Conclusion:**

WPW features familial aggregation and increased arrhythmic risk among siblings of affected individuals despite its extremely low absolute frequency in the general population. The evidence of a measurable hereditary component within an otherwise sporadic, non-syndromic condition points to a genetic contribution driven by complex inheritance patterns.

## Graphical Abstract

**Graphical Abstract:**
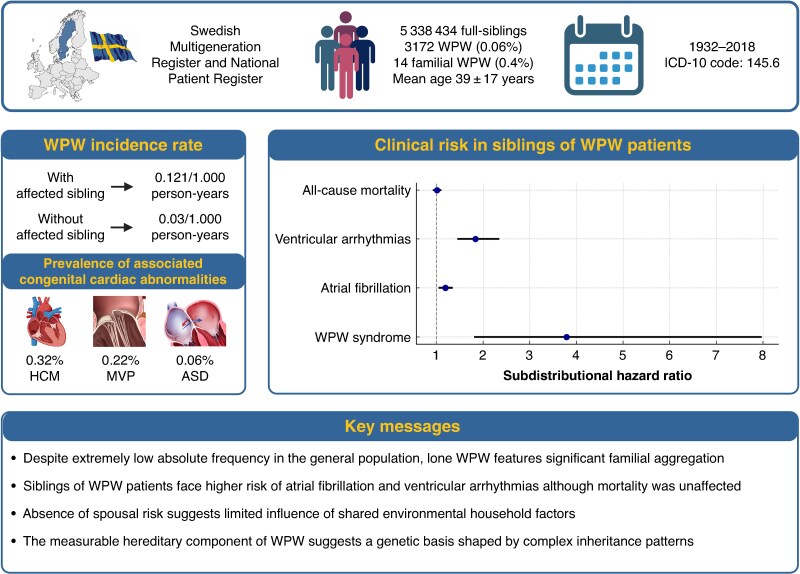

What’s new?

- In this nationwide sibling cohort of 5 338 434 individuals, familial aggregation of WPW syndrome was observed despite its exceptionally low absolute frequency in the general population.
- Among 3172 individuals with WPW (0.06%), only 14 cases (0.4%)—none of which were syndromic—occurred among siblings of affected individuals, corresponding to an absolute frequency of ∼0.0003% in the total population.
- Siblings of affected individuals had a markedly higher rate of WPW (0.121 vs. 0.032 per 1000 person-years), representing nearly a four-fold increase compared with the background population.
- Familial WPW was associated with higher risks of atrial fibrillation and ventricular arrhythmia, although absolute event rates remained low and mortality was unaffected.
- The evidence of a measurable hereditary component in WPW—within its largely sporadic and non-syndromic presentation—indicates that genetic factors may act through complex inheritance patterns.

## Introduction

Wolff–Parkinson–White (WPW) syndrome, first described in 1930, is a rare cardiac conduction disorder characterized by ventricular pre-excitation due to one or more accessory atrioventricular pathways. These pathways bypass the normal conduction system, leading to a shortened PR interval, delta waves on surface electrocardiogram, and increased susceptibility to supraventricular arrhythmias. While the clinical prognosis for most individuals is generally favourable, WPW syndrome poses a higher lifetime risk of malignant ventricular arrhythmias^1^ and sudden cardiac death,^2^ particularly in the context of atrial fibrillation (AF) with rapid ventricular response.^3,4^

WPW affects ∼1.5–3.1 per 1000 individuals, with most cases being sporadic.^3,5^ However, familial clustering has been reported, raising questions about its genetic basis. Familial cases of WPW have been documented, often showing autosomal dominant inheritance^6^ with variable penetrance.^5^ Mutations in the PRKAG2 gene, which encodes the γ2 subunit of AMP-activated protein kinase, have provided further insights into familial WPW, particularly in cases involving hypertrophic cardiomyopathy and glycogen storage disorders.^7^ Nonetheless, the genetic mechanisms underlying non-syndromic WPW cases remain poorly understood.^5,8^

Emerging evidence highlights the interplay between genetic predispositions and environmental factors in WPW, supporting a complex inheritance model involving multiple genes and modifiers rather than simple Mendelian patterns.^5,8^ Familial aggregation of WPW and associated arrhythmias, such as AF and ventricular arrhythmias, underscores the need for systematic investigations into hereditary risks and their clinical implications.^9^

Family risk studies in large samples are instrumental in uncovering patterns of inheritance and disentangling genetic and environmental contribution; however, despite their potential, large population-based family cohort studies remain underutilized.^10^ By comparing disease risks in siblings and spouses with and without affected family members, these studies provide insights into the relative roles of heritability and shared environmental factors.^11,12^ For rare conditions like WPW, familial aggregation offers opportunities to inform clinical management and genetic counselling.^8^

This nationwide family study aims to address the gaps in understanding familial risks associated with WPW syndrome. Leveraging comprehensive data from Swedish national multigenerational registers, we aimed to investigate the familial risk of WPW, among first-degree relatives of affected individuals compared with individuals of unaffected siblings as the reference group, as well as the family risk of AF, ventricular arrhythmias, and all-cause death associated with sibling history of WPW.

## Methods

### Study population

Patients were not involved in the design, conduct, reporting, or dissemination plans of our research. All data were provided by Statistics Sweden and the National Board of Health and Welfare for research purposes. Data were coded according to the European Union law.

The Regional Ethical Review Board in Lund, Sweden, approved this register study, and informed consent was not a requirement due to the nationwide nature of the data. This study followed the ‘Strengthening the Reporting of Observational Studies in Epidemiology’ reporting guideline. We used the following Swedish national registers^13–17^ for data extraction: the Swedish Multigeneration Register, which contains data on familial relationships and index persons born in 1932 and later and registered in Sweden in 1961 and later; the National Patient Register (NPR), which includes hospital discharge diagnoses from 1964 to 2018 with nationwide coverage from 1987, and hospital outpatient diagnoses from 2001 to 2018; the population register and the total population register, which contain data on death date, if applicable, marital status, family relationships, education, and migration (the register has high coverage for nearly 100% of birth and death dates, 95% of immigration events and 91% of emigration events); and the Swedish Cause of Death Register, which provides date and cause of death from 1961 to 2018. The databases were linked together according to previously applied methods.^18,19^

In the Swedish Multigeneration National Swedish Register, we identified all pairs of full-siblings born in Sweden by Swedish-born parents. Thus, both biological parents were obligatorily known. Relative pairs with members who died or emigrated before 1997 or emigrated before the age of 17 years were excluded. Twins were included in the full-sibling group, and no information regarding zygosity was available. Our analysis relied on the International Statistical Classification of Diseases and Related Health Problems, Tenth Revision (ICD-10) code I45.6 to identify WPW syndrome, as earlier ICD versions did not assign a unique diagnosis code for this condition. As a result, cases were restricted to those registered in the NPR between 1997 and 2018. In the database, all relative pairs were double-entered (i.e. all full-sibling pairs as previously described).^18,19^ We allowed the same person to be included in more than one sibling relationship.

### Statistical analysis

The primary exposure was having a sibling diagnosed with WPW. Additional analyses included the familial risk of AF (AF, ICD-10 code I48), ventricular arrhythmias (VAs, ICD-10 codes I47.1 or I49.0), and all-cause mortality. Incidence rates were defined as the number of events divided by the person-time at risk. The familial incidence ratio between two incidence densities (rate in the exposed population divided by rate in those unexposed) gave the incidence rate ratio (IRR). Diagnosed WPW syndrome-free survival curves—as the time to the first recorded WPW diagnosis—were constructed according to the Kaplan–Meier method to compare individuals with and without a documented sibling history of WPW syndrome. For the comparison of two curves, the log-rank test, resulting in a test statistic with a *χ*^2^ distribution and 1 df, was used. The ambiguity in unselected samples as to which sibling’s trait should be used as the dependent, and which as the independent variable, is frequently resolved by using double entry. Each sibling is entered twice in the data, and each member of a sibling pair provides once the dependent and once the explanatory variable. While the consistency of the regression estimates for heritability and environmental influences is not affected by double entry, the standard errors (SEs) of the coefficients are biased and need to be adjusted.^20^ In this study, adjusted familial associations between the full siblings were investigated using the Cox proportional hazards model and were estimated using robust SEs. Results are reported as familial subdistributional hazard ratios (SHRs) with 95% CIs using Fine and Gray’s competing risks model to account for the competing risk of death. Familial HRs for diagnosed WPW syndrome, arrhythmias, and mortality were calculated for siblings of individuals who had a diagnosis of WPW syndrome compared with siblings of individuals unaffected by WPW syndrome as the reference group. Models were adjusted for year of birth, sex, level of education, hypertrophic cardiomyopathy (I42.1–I42.2), mitral valve prolapse (I34.1), Ebstein’s anomaly (Q22.5), and atrial septal defect (Q21.1). We additionally performed a sensitivity analysis to rule out syndromic cases by excluding individuals with hypertrophic cardiomyopathy or congenital conditions including mitral valve prolapse, Ebstein’s anomaly, or atrial septal defect. Sensitivity analyses further excluded cases without procedural confirmation (ICD-10 procedure codes: AF033, DF001, DF002, DF003, DF004, FPB10, FPB12, FPB20, FPB22, FPB32, FPB96, FPC00, FPC10, FPC96, FPD00, FPD96) of WPW and included stratifications by sex, year of birth, and comorbidity (I42.1–I42.2, I34.1, Q21.1) status. Spousal risk was also determined; spouses of WPW cases were used as a negative control group to differentiate genetic from shared environmental influences.^21,22^ Statistical significance was set at *P* < 0.05, and all tests were two-tailed. Data were analysed using SAS V.9.4 (SAS Institute, Cary, NC, USA).

## Results

### Study population

The total study cohort comprised 5 338 434 full-siblings born in Sweden between 1932 and 2018. Overall, 3172 (0.06%) unique individuals were diagnosed with WPW syndrome. WPW was more common among males (60.6%) compared to females. The mean age at WPW diagnosis was 39 ± 17 years. Among WPW patients, education levels varied, with the highest proportion in the 9–11-year group (44.8%), followed by those with >11 years (41.2%), while only 14% had <9 years of education. Cardiovascular comorbidities among WPW probands were few and included hypertrophic cardiomyopathy (0.32%), mitral valve prolapse (0.22%), and atrial septal defect (0.06%) (*Table 1*).

**Table 1:** Characteristics of patients with and without WPW syndrome in the full-siblings study sample

	All individual	No WPW	WPW
	*n* = 5 338 434 (100%)	*n* = 5 335 262 (99.94%)	*n* = 3 172 (0. 06%)
Sex			
Male	2 736 688 (51.26)	2 734 766 (51.26)	1 922 (60.59)
Female	2 601 746 (48.74)	2 600 496 (48.74)	1 250 (39.41)
Education			
Unknown or <9 years	673 471 (12.62)	673 027 (12.61)	444 (14.00)
9–11 years	2 352 221 (44.06)	2 350 800 (44.06)	1 421 (44.80)
> 11 years	2 312 742 (43.32)	2 311 435 (43.32)	1 307 (41.20)
Year of birth			
Mean (SD)	1973 (22)	1973 (22)	1968 (18)
Median (IQR)	1972 (1954–1991)	1972 (1954–1991)	1967 (1953–1982)
(Range)	(1932–2012)	(1932–2012)	(1932–2012)
Age at end follow-up			
Mean (SD)	45 (21)	45 (21)	39 (17)
Median (IQR)	46 (27–63)	46 (27–63)	39 (24–54)
(Range)	(0–86)	(0–86)	(0–83)
Age at WPW onset			
Mean (SD)	NA	NA	39 (17)
Median (IQR)	NA	NA	39 (24–54)
(Range)	NA	NA	(0–83)
Spouse			
Husband	NA	NA	1190 (59.38)
Wife	NA	NA	814 (40.62)
Comorbidities *n*(%)			
Ebstein anomaly	20 (0.00)	19 (0.03)	1 (0.03)
Hypertrophic cardiomyopathy	5 065 (0.09)	5 055 (0.09)	10 (0.32)
Mitral valve prolapse	2 555(0.05)	2 548 (0.05)	7 (0.22)
Atrial septal defect	554 (0.01)	552 (0.01)	2 (0.06)

Age at end of follow-up: death, emigration, diagnoses, or end of study period on 31 December 2018, whichever came first.

SD, standard deviation; IQR, interquartile range; NA, not applicable; WPW, Wolff–Parkinson–White syndrome.

### Familial risk of WPW syndrome

**Figure 1:**
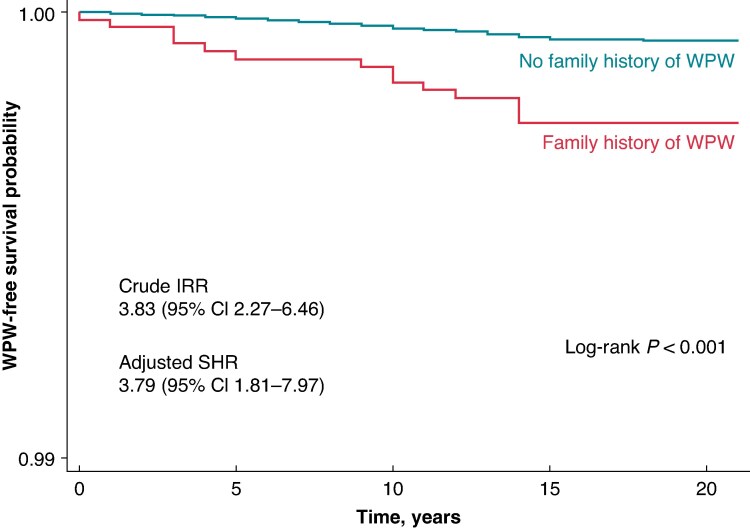
Diagnosed WPW syndrome-free survival estimates by sibling history. Kaplan–Meier estimates of diagnosed WPW syndrome-free survival stratified by sibling history, with crude IRR and subdistribution hazard ratio (SHR) adjusted for sex, birth year, level of education, and congenital heart disease, showing significantly higher risk among siblings of affected individuals.

Overall, only 14 of 3172 cases (0.4%; ≈0.0003% of the total population) were classified as familial. The incidence rate of WPW among individuals with an affected sibling was 0.121 cases per 1000 person-years, compared to 0.032 cases per 1000 person-years in individuals without an affected sibling. Crude IRR for WPW was 3.83 (95% CI 2.27–6.46; *P* < 0.001). Kaplan–Meier survival curves for diagnosed WPW syndrome-free survival showed a significant difference between individuals with and without an affected sibling (log-rank test, *P* < 0.001), with siblings of WPW patients demonstrating lower diagnosed WPW-free survival over time (*Figure 1*). After adjusting for sex, year of birth, education, and comorbidities, sibling history of WPW yielded an subdistribution hazard ratio (SHR) of 3.79 (95% CI 1.81–7.97; *P* < 0.001) (*Table 2*).

**Table 2:** Risk of WPW syndrome by sibling history

Variable	Person-years, No.	Cases, No./persons at risk, No.	Incidence rate, cases/1000 person-years	IRR (95% CI)	^£^SHR(95% CI)		
					Model 1	Model 2	Model 3
Sibling not affected	182 916 443	5 771/9 428 049	0.032 (0.031–0.032)	1 (reference)	1 (reference)	1 (reference)	1 (reference)
Sibling affected	115 972	14/5 785	0.121 (0.071–0.204)	3.83*** (2.27–6.46)	3.87*** (1.84–8.11)	3.81*** (1.81–8.00)	3.79*** (1.81–7.97)

Model 1, crude model; Model 2, adjusted for sex, year of birth, and education; Model 3, adjusted further for comorbidities: hypertrophic cardiomyopathy, mitral valve prolapse, and atrial septal defect. Calculation is based on double entry and ^£^SHRs according to Fine and Gray.CI, confidence interval; IRR, incidence rate ratio; SHR, subdistributional hazard ratio; WPW, Wolff-Parkinson-White.

Significance levels: *P < 0.05, **P < 0.01, ***P < 0.001.

While in the full-cohort analysis examining predictors of WPW, hypertrophic cardiomyopathy (SHR 2.45; 95% CI 1.08–5.55), mitral valve prolapse (2.96; 95% CI 1.20–7.23), and atrial septal defect (5.13; 95% CI 1.29–20.42) were independently associated with a higher likelihood of WPW diagnosis (see Supplementary material online, *Table S1*), all 14 familial WPW cases occurred in the absence of any syndromic condition.

### Spousal analysis

We conducted spousal analysis to assess the extent of environmental sharing in the observed familial risk of WPW syndrome. The incidence rate of WPW was 0.026 cases per 1000 person-years among spouses of affected individuals and 0.032 (0.031–0.032) among spouses of unaffected individuals. No excess risk of WPW syndrome was observed in spouses of affected individuals (adjusted SHR 0.83; 95% CI 0.21–3.31; *P* = 0.76) (see Supplementary material online, *Table S2*).

### Sibling risk of arrhythmic events

Siblings of WPW patients showed a higher incident risk of arrhythmic events compared with siblings of unaffected individuals, regardless of whether they had themselves received a WPW diagnosis or not. The incidence rate of AF among siblings of WPW patients was 2.61 cases per 1000 person-years, compared to 2.21 cases per 1000 person-years in individuals without an affected sibling. Crude IRR for AF was 1.18 (95% CI 1.05–1.32; *P* < 0.01), and the adjusted SHR was 1.19 (95% CI 1.05–1.35; *P* < 0.01) (see Supplementary material online, *Table S3*). The incidence rate of ventricular arrhythmias among siblings of WPW patients was 0.55 cases per 1000 person-years, compared to 0.28 cases per 1000 person-years in individuals without an affected sibling. Crude IRR for ventricular arrhythmias was 1.95 (95% CI 1.53–1.2.49; *P* < 0.001), and the adjusted SHR was 1.84 (95% CI 1.45–2.35; *P* < 0.001) (see Supplementary material online, *Table S4*).

### Sibling risk of all-cause death

All-cause mortality was comparable between individuals with and without a sibling affected by WPW (see Supplementary material online, *Table S5*). The incidence rates of mortality were 3.69 cases per 1000 person-years among siblings of WPW patients and 3.68 cases per 1000 person-years among those without an affected sibling. The adjusted hazard ratio for mortality in siblings of WPW patients was 1.01 (95% CI 0.92–1.11; *P* = 0.88).

### Sensitivity analysis

After exclusion of WPW cases without procedural confirmation, the incidence rate among individuals with affected siblings was 0.105 cases per 1000 person-years, compared to 0.026 cases per 1000 person-years in those without affected siblings. This resulted in an adjusted SHR of 4.07 (95% CI 1.69–9.78; *P* < 0.01) (*Table 3*). In a further sensitivity analysis excluding syndromic cases, the sibling risk of WPW remained significantly elevated (adjusted SHR 3.87; 95% CI 1.84–8.12) (see Supplementary material online, *Table S6*). Excess risk of AF (SHR 1.17; 95% CI 1.03–1.33) and ventricular arrhythmias (SHR 1.89; 95% CI 1.48–2.41) also persisted (see Supplementary material online, *Tables S7* and *S8*), whereas no difference in all-cause mortality was observed (HR 1.00; 95% CI 0.91–1.10) (see Supplementary material online, *Table S9*).

**Table 3:** Risk of WPW syndrome by sibling history: sensitivity analysis with exclusion of WPW cases without procedural confirmation

Variable	Person-years, No.	Cases, No./persons at risk, No.	Incidence rate, cases/1000 person-years	IRR (95% CI)	^£^SHR(95% CI)		
					Model 1	Model 2	Model 3
Sibling not affected	182 906 859	4732/9 427 010	0.026 (0.025–0.027)	1 (reference)	1 (reference)	1 (reference)	1 (reference)
Sibling affected	95 293	10/4742	0.105 (0.056–0.195)	4.05*** (2.18–7.54)	4.10** (1.71–9.86)	4.08** (1.70–9.81)	4.07** (1.69–9.78)

Model 1, crude model; Model 2, adjusted for sex, year of birth, and education; Model 3, adjusted further for comorbidities: hypertrophic cardiomyopathy, mitral valve prolapse, and atrial septal defect. Calculation is based on double entry and ^£^SHRs according to Fine and Gray.CI, confidence interval; IRR, incidence rate ratio; SHR, subdistributional hazard ratio; WPW, Wolff-Parkinson-White.

Significance levels: *P < 0.05, **P < 0.01, ***P < 0.001.

## Discussion

In this nationwide sibling cohort, we found evidence of familial aggregation of WPW syndrome, observed despite its extremely low absolute occurrence in the general population. Only a small proportion of affected individuals had an affected sibling, yet this subgroup exhibited an almost four-fold higher likelihood of diagnosis compared with the background population, suggesting a measurable hereditary contribution within an otherwise largely sporadic condition. This finding aligns with previous observations of autosomal dominant inheritance with incomplete penetrance but also points to more complex inheritance patterns where additional genetic factors, modifier genes, and environmental conditions also shape disease expression.

In the context of complex diseases, it is not uncommon to observe average familial risks of ∼2 in first-degree relatives—a value that, notably, is lower than that observed in the present study. While a familial risk ratio of 2 might initially appear modest, it underscores the utility of investigating both genetic and non-genetic factors contributing to familial aggregation. Indeed, even if the overall familial risk appears moderate, the specific underlying drivers—be they genetic, epigenetic, or environmental—are likely to exert a considerably stronger influence on the observed familial clustering than the aggregate familial risk figure itself. Therefore, it becomes apparent that further dissection of familial risk factors is both warranted and critical for providing deeper insight into disease aetiology and optimizing risk stratification efforts.^23^ The lack of association among spouses with WPW for these biologically unrelated but cohabiting individuals suggests that shared environmental household factors in adulthood may contribute little to familial aggregation for WPW syndrome.^21^

WPW can be broadly categorized into non-syndromic and syndromic.^4^ Non-syndromic WPW constitutes the majority of cases, typically appearing sporadically, though familial cases have been reported.^5^ In contrast, syndromic WPW often occurs alongside congenital conditions, such as Ebstein’s anomaly, hypertrophic cardiomyopathy, metabolic disorders, or mitochondrial syndromes.^7^ Syndromic WPW underscores the genetic heterogeneity of the condition, with PRKAG2-related syndromes exemplifying cases of glycogen storage disease, cardiac hypertrophy, and conduction abnormalities.^7,24^ A critical area of research involves accessory pathway formation in lone WPW, where the search for disease genes should encompass transcription factors regulating AV ring development and genes influencing conduction properties.^3^ Interestingly, none of the familial WPW cases diagnosed in our cohort were linked to syndromic entities, pointing towards intrinsic heritable factors other than secondary congenital or metabolic substrates. Investigating these pathways is key for identifying genetic determinants that may refine genotype–phenotype correlations and contribute to improved clinical risk stratification, guiding both family-based screening strategies and genetic research.^9^

Our data showed higher risks of AF and ventricular arrhythmias in siblings of WPW patients, suggesting potential shared mechanisms that affect cardiac conduction and arrhythmia susceptibility.^25^ The higher incidence of WPW among males and its association with congenital cardiac abnormalities, such as hypertrophic cardiomyopathy and mitral valve prolapse, emphasize the potential interplay between genetic predispositions and developmental factors.^5,26–29^ While the exact mechanism remains unclear, several studies suggest that sex-related differences in conduction system development, cardiac embryology, and hormonal influence on arrhythmia susceptibility may contribute.^30,31^ These observations are consistent with the role of accessory pathway formation during embryogenesis, where disruptions in the atrioventricular junction insulating tissue may lead to ventricular pre-excitation.^30^ A broader view considers whether maternal or paternal inheritance could influence clinical manifestations in affected families, given that certain genetic traits may exhibit parent-of-origin effects.^32^ This line of inquiry adds complexity to the underlying genetics of WPW, where syndromic and non-syndromic forms can overlap. Our findings, which extend beyond known associations with structural cardiac abnormalities,^25^ support the hypothesis that multiple overlapping pathways may be at work. Importantly, after excluding WPW cases associated with structural heart disease, we confirmed that the increased familial risks of WPW syndrome, AF, and ventricular arrhythmias among siblings of WPW patients are independent of syndromic clustering and extend to lone WPW. This reinforces the view that genetic predisposition contributes to WPW expression even in the absence of underlying cardiomyopathies or congenital disorders.

From a clinical perspective, while the absolute prevalence of familial WPW remains very low, our results highlight the need for thorough family history among relatives of WPW patients, particularly when pre-excitation coexists with arrhythmic events. Early recognition of mild or asymptomatic arrhythmias can guide management decisions, including electrophysiologic interventions, medical therapies, or risk-modifying strategies.^33^ Further, familial screening triggered by pre-excitation may improve the timely recognition of conditions that can be challenging to diagnose, including subtle or latent HCM phenocopies that may present with pre-excitation and conduction abnormalities in the absence of overt myocardial hypertrophy.^34^ Indeed, recent studies emphasize how clinical red flags such as pre-excitation can prompt earlier investigation into underlying genetic syndromes.^35^ Genetic counselling may offer guidance in assessing risk of recurrence and shaping targeted surveillance strategies, particularly within syndromic or familial clusters. However, its utility outside these contexts remains limited and should be considered only when clinically justified, not as part of a routine approach. Future endeavours should integrate population-based data with deeper genomic analyses to pinpoint specific variants, modifiers, and pathways that lead to WPW syndrome and may translate into a higher risk of malignant ventricular arrhythmias. Advances in molecular technologies, combined with broad international data collections, hold the promise of enhancing our understanding of how individual genetic profiles interact with external factors.

Our epidemiological findings also resonate with, and indeed reinforce, recent advances in paediatric electrophysiology and the genetics of arrhythmia disorders.^36^ Over the past decades, clinical practice has shifted markedly towards early risk stratification and proactive substrate elimination in children and adolescents with arrhythmic syndromes. This paradigm emphasizes timely diagnosis and early catheter ablation of accessory pathways, such as those underlying WPW, to minimize arrhythmic risk and prevent sudden cardiac death. Simultaneously, significant progress has been achieved in genetic discovery and molecular profiling, moving from the identification of single-gene mutations towards comprehensive genotype–phenotype correlations and personalized management.^37^ Our sibling risk analysis provides epidemiologic context to the evolving understanding of WPW. Despite its exceptional rarity, a nearly four-fold increase in sibling risk—along with higher rates of atrial and ventricular arrhythmias—supports the relevance of family history in clinical evaluation. These findings argue for continued investigation into inherited susceptibility and its clinical implications, while acknowledging that the absolute prevalence of familial WPW remains extremely low.

### Strengths and limitations

A key strength of this study is the availability of comprehensive, population-wide data that enabled reliable linkage of familial relationships and thorough case identification of 5 million individuals providing unparalleled statistical power to detect sibling risk patterns. The large sample size enhances the robustness of our conclusions, and the consistency of sensitivity analysis excluding WPW cases without procedural confirmation bolsters the validity of the observations. However, the absence of direct molecular or genomic information leaves unanswered questions about specific genetic variants or pathways involved in the pathogenesis of WPW syndrome. Additionally, although Swedish population-based registers represent an exceptional resource, the generalizability of these findings may be limited. Diagnostic validity in these registers is high,^15^ with positive predictive values typically ranging from 85% to 95%, particularly for procedure codes^38^ and arrhythmic disorders,^39^ yet results may not fully extend to other ethnic or healthcare settings. As with all observational data, causality cannot be definitively established, and residual confounding cannot be entirely excluded. Finally, surveillance bias may reinforce the observed familial pattern; once a single family member is diagnosed, other relatives are likely to be assessed more intensively, leading to a higher detection rate of subclinical arrhythmias. Distinguishing this detection bias from true heritable risk appears relevant for accurate familial risk estimates. We have not investigated self-reported family history of WPW, which is a limitation regarding the clinical usefulness of sibling history of WPW. Finally, the association between higher education levels and increased WPW risk is likely due to ascertainment bias. Individuals with more education may have greater healthcare access, higher health literacy, and more frequent medical evaluations, leading to increased detection.^40^ Education may also act as a proxy for broader socioeconomic and healthcare-seeking factors not fully accounted for in our analysis. While we adjusted for major comorbidities, residual confounding remains possible. Future studies incorporating healthcare utilization data, including ECG screening rates, could help clarify this association.

## Conclusions

This nationwide sibling study demonstrated a measurable hereditary contribution to WPW syndrome. Siblings of affected individuals had nearly a four-fold higher likelihood of diagnosis and an increased arrhythmic burden, yet familial occurrence was exceptionally rare. These findings indicate that WPW is largely sporadic but may occasionally arise on a background of inherited susceptibility influenced by complex genetic and environmental factors. The results emphasize the clinical value of family history in the evaluation of WPW and call for external validation and continued research integrating genomic and population-level data to improve risk stratification and inform precision management of ventricular pre-excitation and related arrhythmogenic disorders.

## Supplementary Material

euaf285_Supplementary_Data

## Data Availability

The data that support the findings of this study are available from the corresponding author upon reasonable request.
