# Supplementary material for: Familial risk of Wolff–Parkinson–White syndrome: a nationwide family study in Sweden
Source: Europace. 2025 Nov 8;27(11):euaf285. doi: 10.1093/europace/euaf285 (PMC12635823; doi:10.1093/europace/euaf285)
Supplement: euaf285_Supplementary_Data [file euaf285_supplementary_data.docx]

**Familial Risk of Wolff-Parkinson-White Syndrome:**

**a Nationwide Family Study in Sweden**

***Online Supplemental Material***

| Supplemental Table 1. Risk of Wolff-Parkinson-White syndrome:  SHRs for variables included in Model 3 | | | | |
| --- | --- | --- | --- | --- |
| Variables | **Reference** | **SHR** | **95% confidence interval** | |
| Sibling history of WPW | No sibling history | 3.79 | 1.81 | 7.97 |
| Year of birth |  | 0.99 | 0.99 | 0.99 |
| Sex | Male | 0.67 | 0.62 | 0.73 |
| 9-11 y of education | unknown or < 9 | 1.24 | 1.08 | 1.41 |
| >11 y of education | unknown or < 9 | 1.25 | 1.09 | 1.44 |
| Hypertrophic cardiomyopathy | No | 2.45 | 1.08 | 5.55 |
| Mitral valve prolapse | No | 2.96 | 1.20 | 7.23 |
| Atrial septal defect | No | 5.13 | 1.29 | 20.42 |
| Model 3= adjusted for all factors in the table; sibling history, sex, year of birth and education and comorbidities: hypertrophic cardiomyopathy, mitral valve prolapse and atrial septal defect | | | | |

| **Supplemental Table 2. Risk of Wolff-Parkinson-White syndrome in spouses of affected individuals compared with spouses of unaffected individuals** | | | | | | | |
| --- | --- | --- | --- | --- | --- | --- | --- |
| Variable | Person-years,  No. | Cases, No./Persons at risk, No. | Incidence rate, cases/1000 person-years | Incidence rate ratio (95%CI) | ^£^SHR(95% CI) | | |
|  |  |  |  |  | **Model 1** | **Model 2** | **Model 3** |
| Spouse not affected | 182 955 220 | 5 783/9 430 064 | 0.032  (0.031-0.032) | 1 [Reference] | 1 [Reference] | 1 [Reference] | 1 [Reference] |
| Spouse affected | 77 195 | 2/3 770 | 0.026  (0.006-0.104) | 0.82  (0.21-3.28) | 0.85  (0.22-3.33) | 0.83  (0.21-3.31) | 0.83  (0.21-3.31) |
| Model 1 =crude model; Model 2= adjusted for sex, year of birth and education; Model 3 = adjusted further for comorbidities: hypertrophic cardiomyopathy, mitral valve prolapse and atrial septal defect. Calculation is based on double entry and ^£^subdistributional hazard ratios (SHRs) according to Fine and Gray. Significance levels: * p<0.05, ** p<0.01, *** p<0.001. | | | | | | | |

| **Supplemental Table 3. Risk of atrial fibrillation by sibling history of Wolff-Parkinson-White syndrome** | | | | | | | |
| --- | --- | --- | --- | --- | --- | --- | --- |
| Variable | Person-years,  No. | Cases, No./Persons at risk, No. | Incidence rate, cases/1000 person-years | Incidence rate ratio (95%CI) | ^£^SHR(95% CI) | | |
|  |  |  |  |  | **Model 1** | **Model 2** | **Model 3** |
| Sibling not affected | 180 570 516 | 399 171/9 428 049 | 2.21  (2.20-2.22) | 1 [Reference] | 1 [Reference] | 1 [Reference] | 1 [Reference] |
| Sibling affected | 114 168 | 298/5 785 | 2.61  (2.33-2.92) | **1.18****  (1.05-1.32) | **1.17***  (1.03-1.33) | **1.20****  (1.06-1.37) | **1.19****  (1.05-1.35) |
| Model 1 = crude model; Model 2 = adjusted for sex, year of birth and education; Model 3 = adjusted further for comorbidities: hypertrophic cardiomyopathy, mitral valve prolapse and atrial septal defect. Calculation is based on double entry and ^£^subdistributional hazard ratios (SHRs) according to Fine and Gray. Significance levels: * p<0.05, ** p<0.01, *** p<0.001. | | | | | | | |

| **Supplemental Table 4. Risk of ventricular arrhythmia by sibling history of Wolff-Parkinson-White syndrome** | | | | | | | |
| --- | --- | --- | --- | --- | --- | --- | --- |
| Variable | Person-years,  No. | Cases, No./Persons at risk, No. | Incidence rate, cases/1000 person-years | Incidence rate ratio (95%CI) | ^£^SHR(95% CI) | | |
|  |  |  |  |  | **Model 1** | **Model 2** | **Model 3** |
| Sibling not affected | 182 496 647 | 51 871/9 428 049 | 0.28  (0.28-0.29) | 1 [Reference] | 1 [Reference] | 1 [Reference] | 1 [Reference] |
| Sibling affected | 115 466 | 64/5 785 | 0.55  (0.43-0.71) | **1.95*****  (1.53-2.49) | **1.96*****  (1.54-2.49) | **1.87*****  (1.47-2.38) | **1.84*****  (1.45-2.35) |
| Model 1 = crude model; Model 2 = adjusted for sex, year of birth and education; Model 3 = adjusted further for comorbidities: hypertrophic cardiomyopathy, mitral valve prolapse and atrial septal defect. Calculation is based on double entry and ^£^subdistributional hazard ratios (SHRs) according to Fine and Gray. Significance levels: * p<0.05, ** p<0.01, *** p<0.001. | | | | | | | |

| **Supplemental Table 5. Risk of all-cause death by sibling history of Wolff-Parkinson-White syndrome** | | | | | | | | |  |
| --- | --- | --- | --- | --- | --- | --- | --- | --- | --- |
| Variable | Person-years,  No. | Cases, No./Persons at risk, No. | Incidence rate, cases/1000 person-years | Incidence rate ratio (95%CI) | HR(95% CI) | | | |  |
|  |  |  |  |  | **Model 1** | **Model 2** | | **Model 3** | |
| Sibling not affected | 182 916 506 | 672 614/9 428 056 | 3.68  (3.67-3.69) | 1 [Reference] | 1 [Reference] | 1 [Reference] | 1 [Reference] | |  |
| Sibling affected | 115 972 | 428/5 785 | 3.69  (3.36-4.06) | 1.00  (0.91-1.10) | 1.00  (0.90-1.11) | 1.01  (0.92-1.11) | 1.01  (0.92-1.11) | |  |
| Model 1 = crude model; Model 2 = adjusted for sex, year of birth and education; Model 3 = adjusted further for comorbidities: hypertrophic cardiomyopathy, mitral valve prolapse and atrial septal defect. Calculation is based on double entry. Significance levels: * p<0.05, ** p<0.01, *** p<0.001. | | | | | | | | |  |

| **Supplementary Table 6. Risk of Wolff-Parkinson-White syndrome by sibling history of WPW after excluding HCM (I42.1–I42.2), MVP (I34.1), Ebstein (Q22.5) or ASD (Q21.1)** | | | | | | |
| --- | --- | --- | --- | --- | --- | --- |
| Variable | Person-years,  No. | Cases, No./Persons at risk, No. | Incidence rate, cases/1000 person-years | Incidence rate ratio (95%CI) | ^£^SHR(95% CI) | |
|  |  |  |  |  | **Model 1** | **Model 2** |
| Sibling not affected | 182 290 778 | 5 717/9 396 115 | 0.03  (0.03-0.03) | 1 [Reference] | 1 [Reference] | 1 [Reference] |
| Sibling affected | 114 920 | 14/5 731 | 0.12  (0.07-0.20) | **3.88*****  (2.30-6.56) | **3.93*****  (1.87-8.24) | **3.87*****  (1.84-8.12) |
| Model 1 =crude model; Model 2= adjusted for sex, year of birth and education. Calculation is based on double entry and ^£^subdistributional hazard ratios (SHRs) according to Fine and Gray. Significance levels: * p<0.05, ** p<0.01, *** p<0.001. | | | | | | |

| **Supplementary Table 7. Risk of atrial fibrillation by sibling history of Wolff-Parkinson-White syndrome after excluding HCM (I42.1–I42.2), MVP (I34.1), Ebstein (Q22.5) or ASD (Q21.1)** | | | | | | |
| --- | --- | --- | --- | --- | --- | --- |
| Variable | Person-years,  No. | Cases, No./Persons at risk, No. | Incidence rate, cases/1000 person-years | Incidence rate ratio (95%CI) | ^£^SHR(95% CI) | |
|  |  |  |  |  | **Model 1** | **Model 2** |
| Sibling not affected | 180 003 897 | 390 681 / 9 396 115 | 2.17 (2.16-2.18) | 1 [Reference] | 1 [Reference] | 1 [Reference] |
| Sibling affected | 113 298 | 282 /5 731 | 2.49 (2.21-2.80) | **1.15***  (1.02-1.29) | 1.13  (0.99-1.29) | **1.17***  (1.03-1.33) |
| Model 1 =crude model; Model 2= adjusted for sex, year of birth and education. Calculation is based on double entry and ^£^subdistributional hazard ratios (SHRs) according to Fine and Gray. Significance levels: * p<0.05, ** p<0.01, *** p<0.001. | | | | | | |

| **Supplementary Table 8. Risk of ventricular arrhythmia by sibling history of Wolff-Parkinson-White syndrome after excluding HCM (I42.1–I42.2), MVP (I34.1), Ebstein (Q22.5) or ASD (Q21.1)** | | | | | | |
| --- | --- | --- | --- | --- | --- | --- |
| Variable | Person-years,  No. | Cases, No./Persons at risk, No. | Incidence rate, cases/1000 person-years | Incidence rate ratio (95%CI) | ^£^SHR(95% CI) | |
|  |  |  |  |  | **Model 1** | **Model 2** |
| Sibling not affected | 181 879 115 | 50 829/9 396 115 | 0.28  (0.28-0.28) | 1 [Reference] | 1 [Reference] | 1 [Reference] |
| Sibling affected | 114 424 | 63/5 731 | 0.55  (0.43-0.70) | **1.97*****  (1.54-2.52) | **1.97*****  (1.55-2.52) | **1.89*****  (1.48-2.41) |
| Model 1 =crude model; Model 2= adjusted for sex, year of birth and education. Calculation is based on double entry and ^£^subdistributional hazard ratios (SHRs) according to Fine and Gray. Significance levels: * p<0.05, ** p<0.01, *** p<0.001. | | | | | | |

| **Supplementary Table 9. Family risk of all-cause death by sibling history of Wolff-Parkinson-White syndrome after excluding HCM (I42.1–I42.2), MVP (I34.1), Ebstein (Q22.5) or ASD (Q21.1)** | | | | | | |
| --- | --- | --- | --- | --- | --- | --- |
| Variable | Person-years,  No. | Cases, No./Persons at risk, No. | Incidence rate, cases/1000 person-years | Incidence rate ratio (95%CI) | HR(95% CI) | |
|  |  |  |  |  | **Model 1** | **Model 2** |
| Sibling not affected | 182 290 723 | 666 597/9 396 115 | 3.66  (3.64-3.67) | 1 [Reference] | 1 [Reference] | 1 [Reference] |
| Sibling affected | 114 920 | 418/5 731 | 3.64  (3.30-4.00) | 0.99  (0.90-1.10) | 0.98  (0.88-1.09) | 1.00  (0.91-1.10) |
| Model 1 =crude model; Model 2= adjusted for sex, year of birth and education. Calculation is based on double entry.  Significance levels: * p<0.05, ** p<0.01, *** p<0.001. | | | | | | |
